# Characterisation of *CDKL5* Transcript Isoforms in Human and Mouse

**DOI:** 10.1371/journal.pone.0157758

**Published:** 2016-06-17

**Authors:** Ralph D. Hector, Owen Dando, Nicoletta Landsberger, Charlotte Kilstrup-Nielsen, Peter C. Kind, Mark E. S. Bailey, Stuart R. Cobb

**Affiliations:** 1 Institute of Neuroscience and Psychology, College of Medical, Veterinary and Life Sciences, University of Glasgow, Glasgow, United Kingdom; 2 Patrick Wild Centre, University of Edinburgh, Edinburgh, United Kingdom; 3 Centre for Integrative Physiology, University of Edinburgh, Edinburgh, United Kingdom; 4 Centre for Brain Development and Repair, The Institute for Stem Cell Biology and Regenerative Medicine, Bangalore, India; 5 Department of Medical Biotechnology and Translational Medicine, University of Milan, Milan, Italy; 6 Department of Theoretical and Applied Sciences, Division of Biomedical Research, University of Insubria, Busto Arsizio, Italy; 7 School of Life Sciences, College of Medical, Veterinary and Life Sciences, University of Glasgow, Glasgow, United Kingdom; University of Valencia, SPAIN

## Abstract

Mutations in the X-linked Cyclin-Dependent Kinase-Like 5 gene (*CDKL5*) cause early onset infantile spasms and subsequent severe developmental delay in affected children. Deleterious mutations have been reported to occur throughout the *CDKL5* coding region. Several studies point to a complex *CDKL5* gene structure in terms of exon usage and transcript expression. Improvements in molecular diagnosis and more extensive research into the neurobiology of *CDKL5* and pathophysiology of CDKL5 disorders necessitate an updated analysis of the gene. In this study, we have analysed human and mouse *CDKL5* transcript patterns both bioinformatically and experimentally. We have characterised the predominant brain isoform of *CDKL5*, a 9.7 kb transcript comprised of 18 exons with a large 6.6 kb 3’-untranslated region (UTR), which we name *hCDKL5_1*. In addition we describe new exonic regions and a range of novel splice and UTR isoforms. This has enabled the description of an updated gene model in both species and a standardised nomenclature system for *CDKL5* transcripts. Profiling revealed tissue- and brain development stage-specific differences in expression between transcript isoforms. These findings provide an essential backdrop for the diagnosis of CDKL5-related disorders, for investigations into the basic biology of this gene and its protein products, and for the rational design of gene-based and molecular therapies for these disorders.

## Introduction

Mutations in the X-linked Cyclin-Dependent Kinase-Like 5 gene, *CDKL5* (Mendelian Inheritance in Man, MIM: 300203; previously known as *STK9*), cause a range of phenotypes, including EIEE2 (MIM: 300672), a form of early infantile epileptic encephalopathy [1, 2], and infantile spasms [3–7]. *CDKL5* mutations have also been described in patients diagnosed with West syndrome, Lennox-Gastaut syndrome and atypical forms of Rett syndrome (RTT) [4, 5]. In addition to the characteristic early-onset seizures, the phenotype may also include a number of other features, such as stereotypic hand movements, severe psychomotor retardation and general hypotonia [3, 5, 8–11]. Studies also report visual impairment and poor eye contact [5, 9, 10]. It is widely assumed that most *CDKL5*-related phenotypes result from loss of function mutations, but putative gain-of-function mutations [12], as well as large duplication events that include the *CDKL5* gene region, have also been identified [13–15].

*CDKL5* is located on the X-chromosome (Xp22.13) and is subject to X-chromosome inactivation. All reported mutations thus far have dominant effects on phenotype and affected patients are almost always heterozygous females. Hemizygous males are known, however, and are invariably severely affected [6]. The clinical variability observed in heterozygous females may partly be explained by variable X-inactivation ratios in the brain [16]. Incidence data are not widely available, but, altogether, the CDKL5 disorders are becoming recognised as being more common than hitherto appreciated.

Although the CDKL5 disorders are not congenital, the early postnatal onset of symptoms indicates that CDKL5 plays a crucial role in brain development. CDKL5 is also expressed within the mature adult nervous system [12], where it appears especially abundant in forebrain neurons [17]. CDKL5 is expressed throughout the cell, including the nucleus and the cytoplasm of the cell soma and dendrites. Studies in cell and animal models have shown that CDKL5 is important for neurite outgrowth and dendritic spine development as well as for functional neuronal plasticity [18–20]. It has also been implicated as an important regulator of cellular responses to oxidative stress [21]. Two *Cdkl5* knockout (KO) mouse models have been reported, with phenotypes including impaired motor control, altered behaviour, abnormal eye tracking, general hypoactivity and morphological features such as reduced dendritic arborisation of cortical neurons [22, 23]. However, spontaneous seizures, a key feature of CDKL5 disorder in patients, are not observed in mice.

The multiple cellular locations and potential functions of CDKL5 may, in part, be explained by its molecular heterogeneity. CDKL5 is known to exist as a number of isoforms resulting from alternative splicing, and several transcripts have been identified; however, nothing is known about their functional differences or developmental expression. *Cdkl5* transcripts are expressed widely throughout the body [24, 25]. All known CDKL5 isoforms appear to include a highly conserved serine/threonine kinase domain [26] located in the N-terminal half of the protein and a Thr-Xaa-Tyr (TEY) motif that is thought to activate other kinases, such as those in the MAP kinase family [27, 28]. Other functional regions, such as nuclear localisation signals (NLS) and nuclear export signals (NES), are predicted to be present within the otherwise much more poorly characterised C-terminal half of the protein [12]. Loss-of-function mutations are known to occur throughout the coding regions of the gene but many pathogenic missense mutations cluster within the region encompassing the catalytic domain [17, 29].

Knowledge of gene transcript isoforms and their developmental expression profiles is essential for understanding their mechanistic roles in brain development. For example, different isoforms of the *Syngap* gene, mutations in which also lead to EIEE [30], have different developmental expression profiles and opposing effects on synaptic function [31]. Furthermore, comparison of isoforms across species may give insight into species-specific differences in phenotypes. Finally, knowledge of the spatio-temporal expression patterns of *CDKL5* isoforms will provide information that is essential for the development of therapeutic avenues such as gene and protein replacement. A number of studies have investigated *CDKL5* gene structure and transcript expression in different tissues [5, 24, 25, 32, 33] but the set of transcripts and resulting protein isoforms remains incompletely characterised, and a standardised nomenclature for these isoforms has not been instituted. The aim of this study was to characterise the mRNA products of human *CDKL5* and mouse *Cdkl5* using a combination of bioinformatic analyses and molecular methods, and to predict the protein isoforms translated from each transcript. The study has generated a new model of *CDKL5* gene structure in both species and defines a range of previously uncharacterised transcript isoforms with unique developmental expression profiles. To accommodate these complexities in gene expression, we propose a new nomenclature system for *CDKL5* gene products that encompasses both the range of transcript isoforms and the predicted suite of protein isoforms.

## Materials and Methods

### RNA-seq Data Analysis

The following RNA-seq datasets were analysed in this study: human brain GEO sample ID GSM1173806 [34]; human testis GEO sample ID GSM759517 and ENCODE sample ID ENCSR693GGB [35, 36]; mouse brain GEO sample ID’s GSM1020640, GSM1020649 and GSM1020657 [37]; mouse testis GEO sample ID’s GSM1020648, GSM1020656 and GSM1020665 [37]. Mapping RNA-seq reads to the human and mouse genomes was carried out using the STAR read aligner, version 2.4.2a [38]. To generate STAR genome indices for each species, the following command line was run in each case:

STAR --runMode genomeGenerate --genomeDir <species-index-dir> --genomeFastaFiles <fasta-files> --sjdbGTFfile <gtf-file>

where <species-index-dir> is the directory into which the index files were written, <fasta-files> were FASTA-formatted files containing sequences from the primary assembly of the respective species' genome (Ensembl v80), and <gtf-file> was a GTF-formatted genome annotation file for the respective species (also Ensembl v80). To map a particular set of RNA-seq reads to a species' genome, the following command line was run:

STAR --genomeDir <species-index-dir> --readFilesIn <read-files-1> <read-files-2> --outSAMstrandField intronMotif --twopassMode Basic --outSAMtype BAM SortedByCoordinate

where <species-index-dir> was the directory into which the species' index files were written, and <read-files-1> and <read-files-2> enumerated the FASTQ-formatted files containing paired-end RNA-seq reads for the dataset. STAR was run in two-pass mapping mode to give the most sensitive detection of reads mapping across novel splice junctions. RNA-seq output images were generated using the Integrative Genomics Viewer (IGV) and Sashimi plots [39–41].

### RNA Isolation

Human total RNA isolated from a range of adult tissues, including whole brain (pooled from 4 males aged 21–29) and foetal brain (pooled from 21 spontaneously aborted male and female embryos at 26–40 weeks gestation) was obtained from Clontech (Human Total RNA Master Panel II, Cat. #636643), a widely-used commercial panel sourced with ethical approval based on consent; further approval from the local University of Glasgow ethics committee was not required. Mouse total RNA was isolated from tissues obtained from wild-type C57BL/6 mice using the RNeasy Lipid Tissue Kit (Qiagen), according to the manufacturer’s instructions. Animal samples were collected in accordance with the European Communities Council Directive (86/609/EEC) and with the terms of a project license under the UK Scientific Procedures Act (1986). The quality and quantity of isolated RNA was analysed using the RNA 6000 kit on a 2100 Bioanalyzer (Agilent), and using a Nanodrop 1000 spectrophotometer (Thermo Scientific).

### RT-PCR

Total RNA was reverse transcribed using Superscript III (Life Technologies), according to the manufacturer’s protocol, in 20 μl reactions containing 200 ng of RNA template and 1 μM random hexamers. Reactions were then incubated with 2 units of RNaseH (Life Technologies) at 37°C for 20 min. End-point PCR was performed using Maxima Hot Start Green (Thermo Scientific), according to the manufacturer’s protocol, in 50 μl reactions containing 500 nM gene-specific primers, and products visualised on agarose gels. PCR was performed under the following cycling conditions: an initial denaturation at 95°C for 2 min, then 35 cycles of 95°C for 30 s, 58°C for 30 s and 72°C for 90 s, followed by a final extension of 72°C for 7 min. A list of primers used in the study is provided in S1 Fig.

### Quantitative RT-PCR

First-strand synthesis was performed using the qScript Flex kit (Quanta Bioscience), according to the manufacturer’s protocol, in 20 μl reactions containing 200 ng of total RNA template and 500 nM gene-specific primer or random hexamers. SYBR Green PCR reactions were carried out using the PerfeCTa SYBR kit (Quanta Bioscience), according to the manufacturer’s protocol, in 20 μl reactions using 1/20^th^ of the first-strand synthesis reaction and 300 nM gene-specific primers. PCR was performed under the following cycling conditions on an Mx3005P thermocycler (Agilent Technologies): an initial denaturation at 95°C for 30 s, then 40 cycles of 95°C for 10 s and 60°C for 60 s, followed by a dissociation curve. Appropriate controls were included as recommended by the MIQE guidelines [42]. A list of primers used in the study is provided in S1 Fig. Results were analysed using the comparative quantification analysis tools with MxPro software (Stratagene)

### Rapid Amplification of cDNA Ends (RACE)

3’-RACE was performed using the 3’-RACE System for Rapid Amplification of cDNA ends (Life Technologies), according to the manufacturer’s protocol, in 20 μl reactions containing 200 ng of total RNA template. 5’-RACE was performed using the 5’-RACE System for Rapid Amplification of cDNA ends, Version 2.0 (Life Technologies), according to the manufacturer’s protocol, in 25 μl reactions containing 500 ng of total RNA template and 2.5 pmol of gene-specific primer. Primary and nested PCR reactions were performed using Platinum Taq polymerase (Life Technologies), according to the manufacturer’s protocol, in 50 μl reactions containing 500 nM gene-specific primer. A list of primers used in the study is provided in S1 Fig. PCR products were cloned using the TOPO TA Cloning Kit (Life Technologies) according to the manufacturer’s protocol. Individual colonies were grown in L-Broth, and plasmids were purified using the PureYield Plasmid Miniprep System (Promega) and then sequenced (Source BioScience).

## Results

### Human *CDKL5* Transcript Isoforms

**Fig 1:**
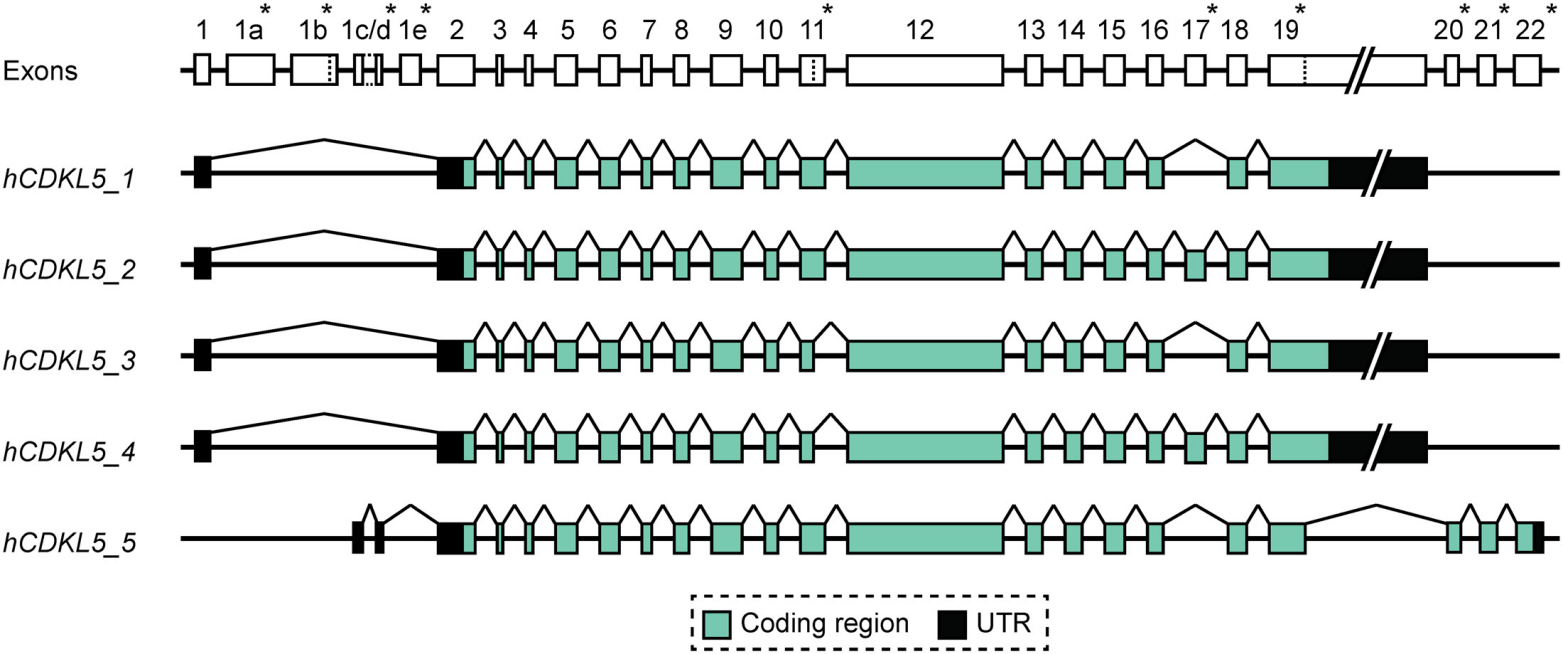
Human *CDKL5* gene and transcript isoforms. Diagram depicting the structure of the human *CDKL5* gene and the exon composition of the five different coding isoforms (*hCDKL5_1* to *hCDKL5_5*). Lines linking exons indicate splicing events. Asterisks next to exon numbers indicate where differences are found between different transcript isoforms. Dotted lines within exons indicate alternative splice sites. Introns and the 3’-UTR portion of exon 19 are not drawn to scale.

To conduct a comprehensive assessment of human *CDKL5* transcripts, including the possibility of novel exons and splicing events, human tissue-specific RNA-seq datasets were analysed to identify all, including very rare, *CDKL5* splicing events. Using sensitive alignment tools to detect reads mapping across all potential splice junctions we detected 27 discrete exons. In addition to confirming the locations of previously identified exon boundaries, we identified novel exons and cryptic splice sites (summarised in Fig 1). The positions of all exon boundaries and splice junctions were validated experimentally by sequencing RT-PCR products spanning multiple exons, and the composition of each specific isoform detected was investigated using isoform-specific RT-PCR assays (design details are given in S1 Fig). The combined RNA-seq and RT-PCR data demonstrated the existence of five major transcript isoforms containing distinct coding regions (with respect to protein coding), which we have termed *hCDKL5_1* to *hCDKL5_5* (Fig 1; see nomenclature recommendations section below). Transcript composition, exon boundaries and chromosomal sequence coordinates are detailed in Table 1.

**Table 1:** Summary of *hCDKL5* transcript isoforms.

Human														
		X-chromosome coordinates		*CDKL5* isoform										
Exon	Size (bp)	Exon start	Exon end	1					2	3	4	5		
1	88	*18425608*	18425695	+	+				+	+	+			
1a^#^	333	*18426069*	18426401			+								
1b^#^	**229**	***18426691***	**18426919**				+							
	**44**	**18426876**	**18426919**		+									
1c	38	*18442224*	18442261									+		
1d	49	18442375	18442423									+		
1cd^#^^$^	200	*18442224*	18442423										+	
1e^#^	**121**	***18443839***	**18443959**					+						+
	**141**	**18443819**	**18443959**										+	
2	226	18506935	18507160	+	+	+	+	+	+	+	+	+	+	+
3	35	18510820	18510854	+	+	+	+	+	+	+	+	+	+	+
4	46	18564477	18564522	+	+	+	+	+	+	+	+	+	+	+
5	137	18575354	18575490	+	+	+	+	+	+	+	+	+	+	+
6	121	18579848	18579968	+	+	+	+	+	+	+	+	+	+	+
7	60	18581891	18581950	+	+	+	+	+	+	+	+	+	+	+
8	91	18584263	18584353	+	+	+	+	+	+	+	+	+	+	+
9	190	18587954	18588143	+	+	+	+	+	+	+	+	+	+	+
10	81	18595348	18595428	+	+	+	+	+	+	+	+	+	+	+
11^#^	**152**	**18598462**	**18598613**	+	+	+	+	+	+			+	+	+
	**101**	**18598462**	**18598562**							+	+			
12	967	18603902	18604868	+	+	+	+	+	+	+	+	+	+	+
13	102	18608811	18608912	+	+	+	+	+	+	+	+	+	+	+
14	106	18609465	18609570	+	+	+	+	+	+	+	+	+	+	+
15	124	18613152	18613275	+	+	+	+	+	+	+	+	+	+	+
16	100	18619867	18619966	+	+	+	+	+	+	+	+	+	+	+
17	123	18623879	18624001						+		+			
18	120	18625128	18625247	+	+	+	+	+	+	+	+	+	+	+
19^#^	**217**	**18628371**	**18628587**									+	+	+
	**7025**	**18628371**	**18635395**	+	+	+	+	+	+	+	+			
	**10037**	**18628371**	**18638408**	+_XL_	+_XL_	+_XL_	+_XL_	+_XL_	+_XL_	+_XL_	+_XL_			
20	84	18646007	18646090									+	+	+
21	183	18650410	18650592									+	+	+
22^#^	199	18653432	18653630									+	+	+

^#^ novel exon, splice site or UTR, not previously characterised

^$^ exon 1cd represents a readthrough of exon 1c to the end of exon 1d

Exon numbers, sizes and co-ordinates for the human *CDKL5* gene (GRCh38/hg38 assembly). Exon composition of each transcript isoform is indicated. Starting coordinates for the main TSS of each initial exon are italicised; coordinates of polyadenylation sites in each terminal exon are underlined. Bold indicate exons that contain multiple splice sites. Note that isoform *hCDKL5*_1 is labelled as having sub-isoforms beginning at each of the alternative first exons, 1a, 1b and 1e; this is also expected to be true for isoforms *hCDKL5_2*, *_3* and *_4*. A subscripted ‘XL’ symbol signifies usage of a more distal polyadenylation signal and consequently the presence of an extended 3’-UTR.

**Fig 2:**
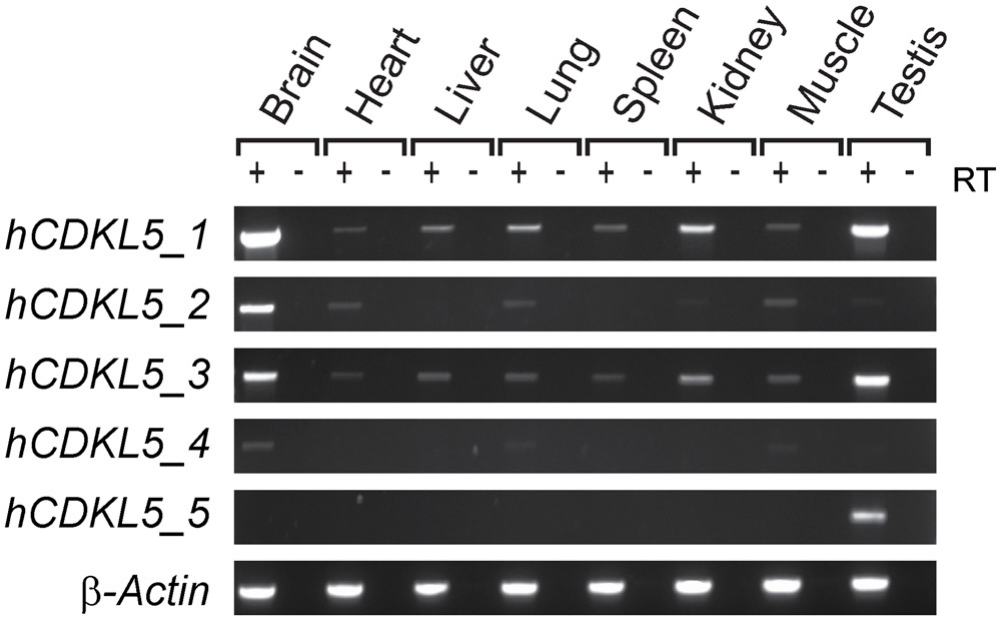
*CDKL5* isoform expression in human tissues. RT-PCR analysis of *CDKL5* isoforms in a panel of adult tissues. β-Actin was used as a loading control.

RT-PCR was carried out to assess transcript expression across a range of tissues (Fig 2). *hCDKL5_1* is the most abundant isoform expressed within the central nervous system (CNS) and was detected in all adult tissues tested, appearing especially abundant in CNS, kidney, testis, prostate gland, thymus and thyroid gland (Fig 2, S2 Fig). This isoform encodes the same protein as the *CDKL5*_107_ transcript reported previously [25]. *hCDKL5_2* is identical to *hCDKL5_1* but also includes exon 17 (previously called 16a or 16b; refs. [33] and [32] respectively), generating a coding sequence 123 bases (41 a.a.) larger than that of *hCDKL5_1*. *hCDKL5_3* and *hCDKL5_4* are identical to *hCDKL5_1* and *hCDKL5_2*, respectively, but lack 51 bases of coding sequence from the 3’ end of exon 11 due to utilisation of a cryptic splice donor site (S3 Fig). The predicted protein made from these transcripts is 17 a.a. shorter than *hCDKL5_1*, or *hCDKL5_2*, respectively. *hCDKL5_2*, *hCDKL5_3* and *hCDKL5_4* are also widely expressed but appear to be of somewhat lower abundance than *hCDKL5_1*. Analysis of read count data in RNA-seq datasets estimates that transcripts incorporating exon 17 (*hCDKL5_*2 and *hCDKL5_*4) constitute approximately 10% of *CDKL5* transcripts expressed in whole brain, while those using the alternative splice site in exon 11 (*hCDKL5_3 and hCDKL5_4*) constitute less than 5% of transcripts in brain (S3 Fig). These observations are supported by semi-quantitative RT-PCR data (Fig 2). In contrast to the first four isoforms, *hCDKL5_5* (previously known as *CDKL5*_*115*_) was only detected in testis amongst the adult tissues tested. These differences in relative abundance are consistent with a previous report [25]. *hCDKL5_5* differs from the other transcripts at the 3’ end by splicing from a cryptic splice site in the newly-named exon 19 to exons 20, 21 and 22 (see Fig 1 and nomenclature section below). We found no evidence for other transcript variants utilising exons 20–22.

**Fig 3:**
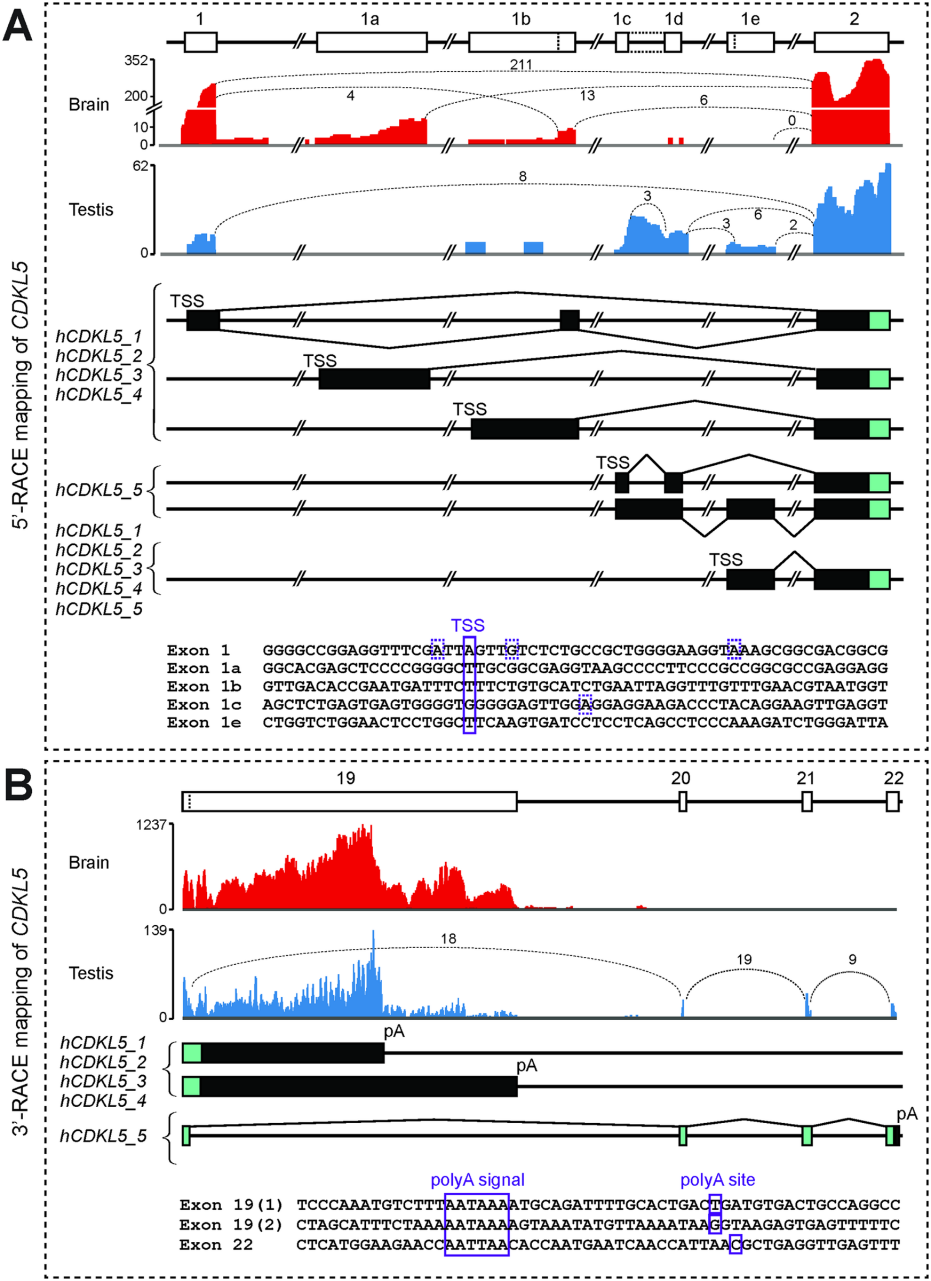
RNA-seq and 5’- and 3’-RACE mapping of human *CDKL5* transcripts. (A) Upper panels: RNA-seq data from brain (red) and testis (blue) datasets show reads mapping to the 5’ end of *CDKL5* (the y-axis indicates read count across the analysed region). Indicative numbers of RNA-seq reads spanning each exon junction are also shown, indicated by values and dotted lines joining exon boundaries. Middle panels: boxes representing each exon at the 5’ end of the gene are shown, aligned with those in the upper panels. Transcription Start Sites (TSSs) and splice events upstream of exon 2 are indicated. Coding regions are indicated by cyan colouring and 5’-UTRs by black colouring. Lower panel: exonic sequences for each first exon are shown. TSSs, confirmed by sequencing of 5’-RACE products, are indicated by boxes; the major TSS is indicated by a solid box, minor TSSs are indicated by hatched boxes. (B) Upper panels: RNA-seq data from brain and testis datasets show reads mapping to the 3’ end of *CDKL5*; exon boundary-spanning read counts are also shown, as in (A), above. Middle panels: the exon composition and splicing patterns at the 3’ end of each human isoform is indicated, colouring as in (A) above. Lower panel: sequences around each of the three polyadenylation signals and sites (pA) are shown; each was confirmed by 3’-RACE mapping.

Analysis of the 5’ region of the gene from RNA-seq and 5’-Rapid Amplification of cDNA Ends (5’-RACE) data revealed multiple transcription start sites (TSSs) (Fig 3A). This indicates the use of five separate initial exons and, thus, promoters. These five initial exons are spaced over an 18 kb region upstream of exon 2. An analysis of potential *CDKL5* open reading frames (ORFs) predicts that all of these different 5’ ends would utilise the same ATG start codon in exon 2, and therefore the different TSSs reflect differences in 5’-untranslated region (UTR) composition only (Fig 3A). Exons 1, 1a and 1b are used in isoforms with notable expression in the adult brain, whereas exons 1c and 1d are used by isoforms expressed in the adult testis. The TSS of Exon 1a was inferred from RNA-seq data and confirmed using a specific RT-PCR assay (Fig 3A; this TSS could not be detected by 5’-RACE, possibly due to unusual RNA secondary structure close to the 5’ end). Exon 1e was found to be present in all five major isoforms (*hCDKL5_1* –*hCDKL5_5*) but was never used in combination with exons 1, 1a or 1b. Alternative splicing and read-through events (such as from exon 1c to 1d in some isoforms) result in a complex set of *CDKL5* 5’-UTRs, described in Fig 3A and Table 1. Only a small number of RNA-seq reads map to exons 1a and 1b in the brain, suggesting that these represent lower abundance isoforms (Fig 3A). Although no RNA-seq reads mapped to exon 1e in the brain sample analysed, RT-PCR and 5’-RACE data confirmed that transcripts initiating from this exon were indeed present in the brain (not shown).

*CDKL5* transcript 3’-UTRs have never been characterised in detail before. Previous northern blot data [24] had suggested that some *CDKL5* transcripts may be extremely large, potentially bigger than 10 kb. Here, we analysed 3’-UTRs using RNA-seq datasets and 3’-RACE to search for potential polyadenylation sites within the predicted UTR regions encoded by exons 19 and 22. We identified two separate canonical polyadenylation signals (AATAAA) in exon 19 used in the *hCDKL5_1*, _*2*, _*3* and _*4* transcripts (Fig 3B). These two signals, located 6.6 kb and 9.7 kb downstream of the stop codon, are predicted to give rise to transcripts of approximately 9.7 and 12.8 kb, respectively. Our RT-PCR analysis indicates that both these long 3’-UTRs are used by each of the *hCDKL5_1*, _*2*, _*3* and _*4* transcripts (data not shown). In the case of the *hCDKL5_5* transcript, the composition of the C-terminal region of the protein and the sequence of the 3’-UTR is entirely different to that of the other isoforms. 3’-RACE identified a non-canonical polyadenylation signal (AATTAA) used by this isoform downstream of the stop codon in exon 22 (Fig 3B), giving rise to a 3’-UTR of 86 bases for this isoform.

### Mouse *Cdkl5* Transcript Isoforms

**Fig 4:**
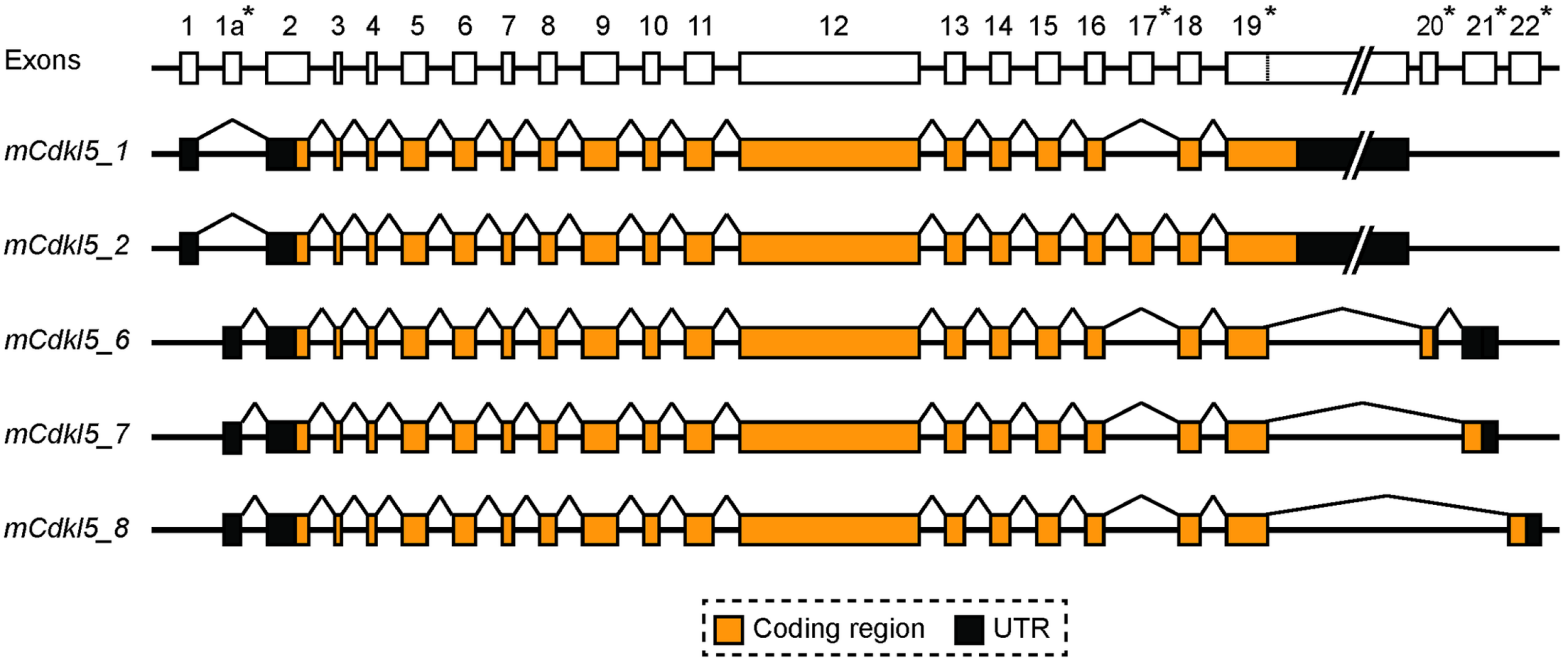
Mouse *Cdkl5* gene and transcript isoforms. Diagram depicting the structure of the mouse *Cdkl5* gene and the exon composition of the five different coding isoforms (*mCDKL5_1*, *_2*, *_6*, *_7*, *_8)*. Lines linking exons indicate splicing events. Asterisks next to exon numbers indicate where differences are found between different transcript isoforms. Dotted lines within exons indicate alternative splice sites. Introns and the 3’-UTR portion of exon 19 are not drawn to scale.

To complement the new analysis of human *CDKL5*, a detailed analysis of mouse *Cdkl5* transcripts was also carried out (summarised in Fig 4). Mouse tissue-specific RNA-seq datasets were analysed using the same methods described above to identify all potential *Cdkl5* splicing events, confirming the existence of previously identified exon boundaries and suggesting the use of novel exons (Fig 4). RT-PCR assays were carried out and all products sequenced to experimentally validate the presence of these splice events in *Cdkl5* transcripts (S1B Fig). Altogether, a total of 23 exons were identified, three of which have not been previously characterised, exons 1a, 20 and 21. Exon boundaries and chromosomal sequence coordinates are detailed in Table 2. Combined, the data demonstrate the existence of five major transcript isoforms containing distinct coding regions (Fig 4). We have termed the first two isoforms *mCdkl5_1* and *mCdkl5_2* as they are orthologous to human isoforms *_1* and *_2*, respectively. In contrast, the coding regions of the other three transcripts do not show full orthology to human isoforms and are hence termed *mCdkl5_6*, *mCdkl5_7* and *mCdkl5_8* (Fig 4).

**Table 2:** Summary of *mCdkl5* transcript isoforms.

Mouse								
		X-chromosome coordinates		*Cdkl5* isoform				
Exon	Size (bp)	Exon start	Exon end	1	2	6	7	8
1	91	*160994688*	160994591	+	+			
1a^#^^$^	200	*160976506*	160976307			+	+	+
2	231	160928655	160928425	+	+	+	+	+
3	35	160924486	160924452	+	+	+	+	+
4	46	160876568	160876523	+	+	+	+	+
5	137	160866053	160865917	+	+	+	+	+
6	121	160861054	160860934	+	+	+	+	+
7	60	160858859	160858800	+	+	+	+	+
8	91	160856445	160856355	+	+	+	+	+
9	190	160852330	160852141	+	+	+	+	+
10	81	160847017	160846937	+	+	+	+	+
11	152	160840446	160840295	+	+	+	+	+
12	967	160834834	160833868	+	+	+	+	+
13	102	160830022	160829921	+	+	+	+	+
14	106	160829648	160829543	+	+	+	+	+
15	124	160824098	160823975	+	+	+	+	+
16	100	160816991	160816892	+	+	+	+	+
17	123	160813050	160812928		+			
18	120	160811716	160811597	+	+	+	+	+
19^#^	**217**	**160808691**	**160808475**			+	+	+
	**7065**	**160808691**	**160801627**	+	+			
20^#^^$^	83	160800092	160800010			+		
21^#^^$^	141	160799251	160799111			+	+	
22^#^^$^	280	160784582	160784582					+

^#^ novel exon or UTR, not previously characterised

^$^ mouse-specific, not orthologous to human

Exon numbers, sizes and co-ordinates for the mouse *Cdkl5* gene (GRCm38/mm10 assembly). Exon composition of each transcript isoform is indicated. Starting coordinates for the main TSS of each initial exon are italicised; coordinates of polyadenylation sites in each terminal exon are underlined. Bold indicate exons that contain multiple splice sites.

**Fig 5:**
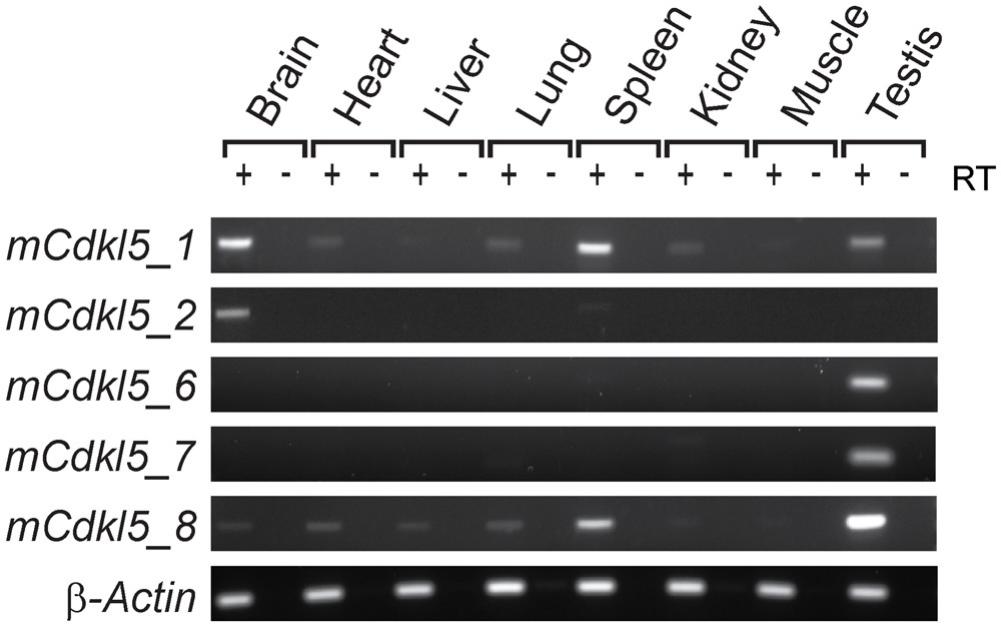
*Cdkl5* isoform expression in mouse tissues. RT-PCR analysis of *Cdkl5* isoforms in a panel of adult tissues. β-Actin was used as a loading control.

New RT-PCR assays capable of differentiating between *Cdkl5* transcript isoforms were developed and used to assess transcript expression across a range of tissues (Fig 5). *mCdkl5_1* is completely orthologous to its human counterpart and also appears to be the most abundant isoform expressed within the mouse brain. It was also detected in a variety of other adult tissues, in agreement with a previous study [25]. We also confirm that *mCdkl5_2* is completely orthologous to its human counterpart. The levels of *mCdkl5_2* expression in the brain appear to be approximately 10% of that of *mCdkl5_1*, according to our analysis of read counts in RNA-seq datasets (S3 Fig). No mouse orthologues of *hCDKL5_3* and *hCDKL5_4* were identified. Sequence analysis of exon 11 revealed that the cryptic splice donor site is absent in mouse due to a single nucleotide difference (S3 Fig). BLAST analysis (http://blast.ncbi.nlm.nih.gov/Blast.cgi) revealed that the cryptic splice site consensus sequence is present in all mammals with *Cdkl5* sequences in Genbank except mouse and a few other rodent species (not shown).

**Fig 6:**
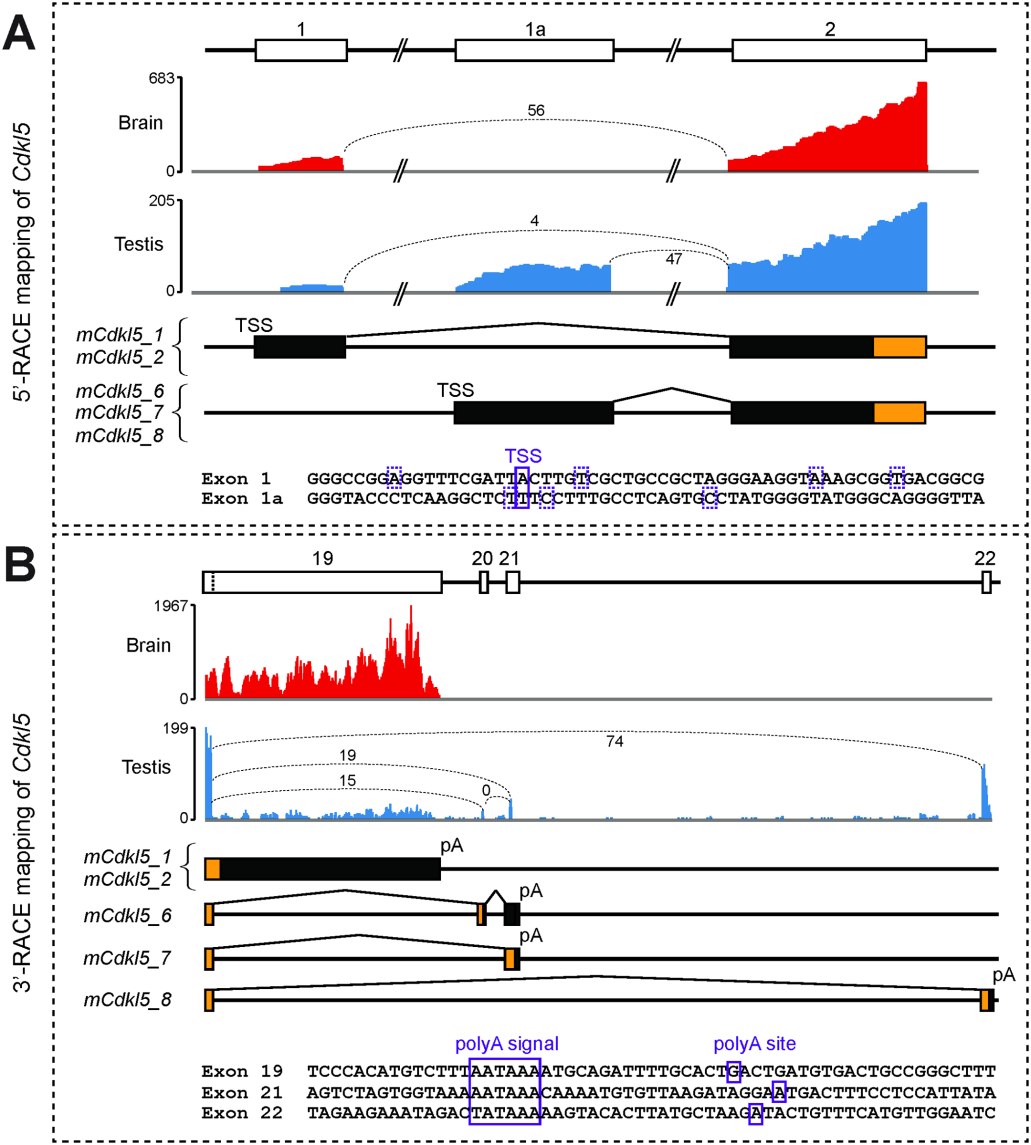
RNA-seq and 5’ and 3’-RACE mapping of mouse *Cdkl5* transcripts. (A) Upper panels: RNA-seq data from brain (red) and testis (blue) datasets show reads mapping to the 5’ end of *Cdkl5* (the y-axis indicates read count across the analysed region). Indicative numbers of RNA-seq reads spanning each exon junction are also shown, indicated by values and dotted lines joining exon boundaries. Middle panels: boxes representing each exon at the 5’ end of the gene are shown, aligned with those in the upper panels. Transcription Start Sites (TSSs) and splice events upstream of exon 2 are indicated. Coding regions are indicated by orange colouring and 5’-UTRs by black colouring. Lower panel: exonic sequences for each first exon are shown. TSSs, confirmed by sequencing of 5’-RACE products, are indicated by boxes; the major TSS is indicated by a solid box, minor TSSs are indicated by hatched boxes. (B) Upper panels: RNA-seq data from brain and testis datasets show reads mapping to the 3’ end of *Cdkl5*; exon boundary-spanning red counts are also shown, as in (A), above. Middle panels: the exon composition and splicing patterns at the 3’ end of each mouse isoform is shown, colouring as in A) above. Lower panel: sequences around each of the three polyadenylation signals and sites (pA) are shown; each was confirmed by sequencing of 3’-RACE mapping.

The alternative splice donor site in exon 19 is also conserved between species, and is used in three of the mouse isoforms (*mCdkl5_6*, *_7* and *_8*). Bioinformatic analysis of RNA-seq datasets indicated that three exons lie downstream of exon 19 (Fig 6), of which only one had previously been reported (Genbank accession: NM_001024624.2). The newly identified exons were named exons 20 and 21, while the previously characterised exon lying approximately 15 kb downstream of exon 21 is renumbered as exon 22. Each of these three exons was confirmed by sequencing of RT-PCR products. Exon boundaries and chromosomal sequence coordinates are detailed in Table 2. BLAST searches revealed that none of these three exons is clearly orthologous to any conserved human or other mammalian genomic sequence downstream of the conserved exon 19, and current evidence suggests that they are therefore specific to mouse. Interestingly, expression of *mCdkl5_6* and *mCdkl5_7*, like that of *hCDKL5_5*, is confined to the testis in the adult mouse, whereas *mCdkl5_8* is expressed in the spleen and at very low levels in other tissues, such as brain, heart, liver and lung, as well as in the testis (Fig 5).

5’-RACE was used to characterise the 5’ end of *Cdkl5*. The major TSS in brain was mapped to position 160994677 (mm10 genomic reference sequence coordinates), indicating usage of exon 1 by transcript isoforms *mCdkl5_1* and *_2* (Fig 6A). Sequencing of multiple 5’-RACE product clones also identified other TSS’s in close proximity (Fig 6A). The 5’-UTR of these transcripts therefore comprises approximately 260 bases. Exon 1 is well conserved between species (sequence identity > 80%) and the sites of the major TSSs in mouse and human are orthologous. 5’-RACE was also used to characterise the 5’ end of *Cdkl5* transcripts that use the novel, mouse-specific exon 1a. RT-PCR analysis indicated that this exon is used as an alternative first exon in isoforms *mCDKL5_6*, *mCDKL5_7* and *mCDKL5_8*, which are predominantly expressed in testis in the adult. Multiple TSSs were identified, clustered closely together at the start of exon 1a (Fig 6A). The major TSS was mapped to position 160976506 (mm10, as above), yielding transcripts with a 5’-UTR of 367 bases (Fig 6A).

3’-RACE analysis confirmed the existence of three alternatively-spliced transcript isoforms that utilise exons 20–22 (Fig 6B). In *mCdkl5_6* the conserved alternative splice donor site in exon 19 splices to exon 20, then to exon 21, where it terminates (Figs 4 and 6B). In *mCdkl5_7*, splicing is from the alternative splice donor site in exon 19 directly to exon 21 (Figs 4 and 6B). Both *mCdkl5_6* and *mCdkl5_7* utilise the same downstream canonical polyadenylation signal, resulting in short 168 base and 58 base 3’-UTRs, respectively (Fig 6B). However, the inclusion of exon 20 in *mCdkl5_6* results in a coding frame that terminates at a stop codon near the end of exon 20. As splicing is detected after this exon in our 3’-RACE assay, it may be that nonsense-mediated decay does not operate to degrade transcripts using this combination of exons. In *mCdkl5_8*, splicing occurs from the alternative splice donor site in exon 19 directly to exon 22, as previously reported (Figs 4 and 6B). This transcript contains a unique, 176 bp 3’-UTR terminating with a non-canonical polyadenylation signal (TATAAA) downstream of the stop codon in exon 22 (Fig 6B).

3’-RACE was also used to characterise the 3’-UTRs of the human-orthologous transcripts *mCdkl5_1* and *mCdkl5_2*. In both cases, a single canonical polyadenylation signal located 6.6 kb downstream of the stop codon in exon 19 was confirmed (Fig 6B). The signal is situated at orthologous positions in each species, and yields transcripts of approximately 9.9 kb in the mouse.

### Nomenclature Recommendations

Based on the work described above, a new nomenclature system for human *CDKL5* and mouse *Cdkl5* transcript isoforms is proposed here (Table 3). For each transcript, the gene symbol is italicised; *CDKL5* for human, *Cdkl5* for mouse. This may be preceded by a letter to indicate the species; *h* for human, *m* for mouse. This allows the naming of transcripts from other species, such as rat, (indicated by an *r*), within the proposed system. The gene symbol is followed by an underscore and a number, which will differentiate transcripts with different coding sequences. The system is based on orthology, so the same number in different species indicates an orthologous coding sequence; for example, *hCDKL5_1* comprises exonic sequences that seem to be entirely orthologous to those found in *mCdkl5_1*. The proteins made from each transcript will be named according to the same convention i.e. transcript *hCDKL5_1* encodes protein isoform hCDKL5_1.

**Table 3:** Summary of *CDKL5*/*Cdkl5* nomenclature.

New isoform nomenclature		Coding region sizes				
Human	Mouse	(bp)	(aa)	Former name	Ensembl transcript	Descriptive name
*hCDKL5_1*	*mCdkl5_1*	2883	960	CDKL5_107_	CDKL5-005/Cdkl5-003	*hCDKL5_2–19Δ17*
*hCDKL5_2*	*mCdkl5_2*	3006	1001	CDKL5-16b	-	*hCDKL5_2–19*
*hCDKL5_3*	-	2832	943	-	-	*hCDKL5_2-19s11Δ17*
*hCDKL5_4*	-	2955	984	-	-	*hCDKL5_2-19s11*
*hCDKL5_5*	-	3093	1030	CDKL5_115_	CDKL5-002	*hCDKL5_2–22 Δ17s19*
-	*mCdkl5_6*	2769	923	-	-	*mCdkl5_2–21 Δ17s19*
-	*mCdkl5_7*	2796	931	-	-	*mCdkl5_2–21 Δ17s19Δ20*
-	*mCdkl5_8*	2817	938	CDKL5_105_	Cdkl5-001	*mCdkl5_2–22 Δ17s19Δ20Δ21*

The new proposed nomenclature system, former names, equivalent Ensembl transcript model and coding region sizes. The descriptive name indicates, in addition to the major coding isoform, the variable exon usage associated with each transcript: Δ indicates that an exon is not used; s indicates that the short form of the exon is used, where an alternative splice site is present.

At the 5’ end of *CDKL5* isoforms expressed in the brain, most transcripts splice from exon 1 directly to exon 2 (Fig 3A). However, the identification of novel exons, TSSs and alternative splicing in this region has shown that multiple minor transcripts display variation in their 5’-UTRs, but not in their ORFs (Fig 3A). Additional variation is also seen at the 5’ end of *hCDKL5_5* (see Table 1 for a description of this variation). At the 3’ end, RACE mapping has identified unique polyadenylation signals and 3’-UTRs for most transcripts utilising the different final exons in *CDKL5* and *Cdkl5* (Figs 3B and 6B). However, an exception occurs with transcripts terminating at exon 19 in human, where some transcripts use a more distal polyadenylation signal, resulting in a larger 3’-UTR and a longer transcript (~12.8 kb). To distinguish the extra-long (and less abundant) ~12.8 kb transcripts from the abundant 9.7kb transcripts, a subscripted ‘*XL*’ symbol at the end of the name signifies usage of the distal polyadenylation signal and inclusion of the extra-long 3’UTR i.e. *hCDKL5_1*_*XL*_ (Table 1).

### Expression of *CDKL5*/*Cdkl5* during Development

**Fig 7:**
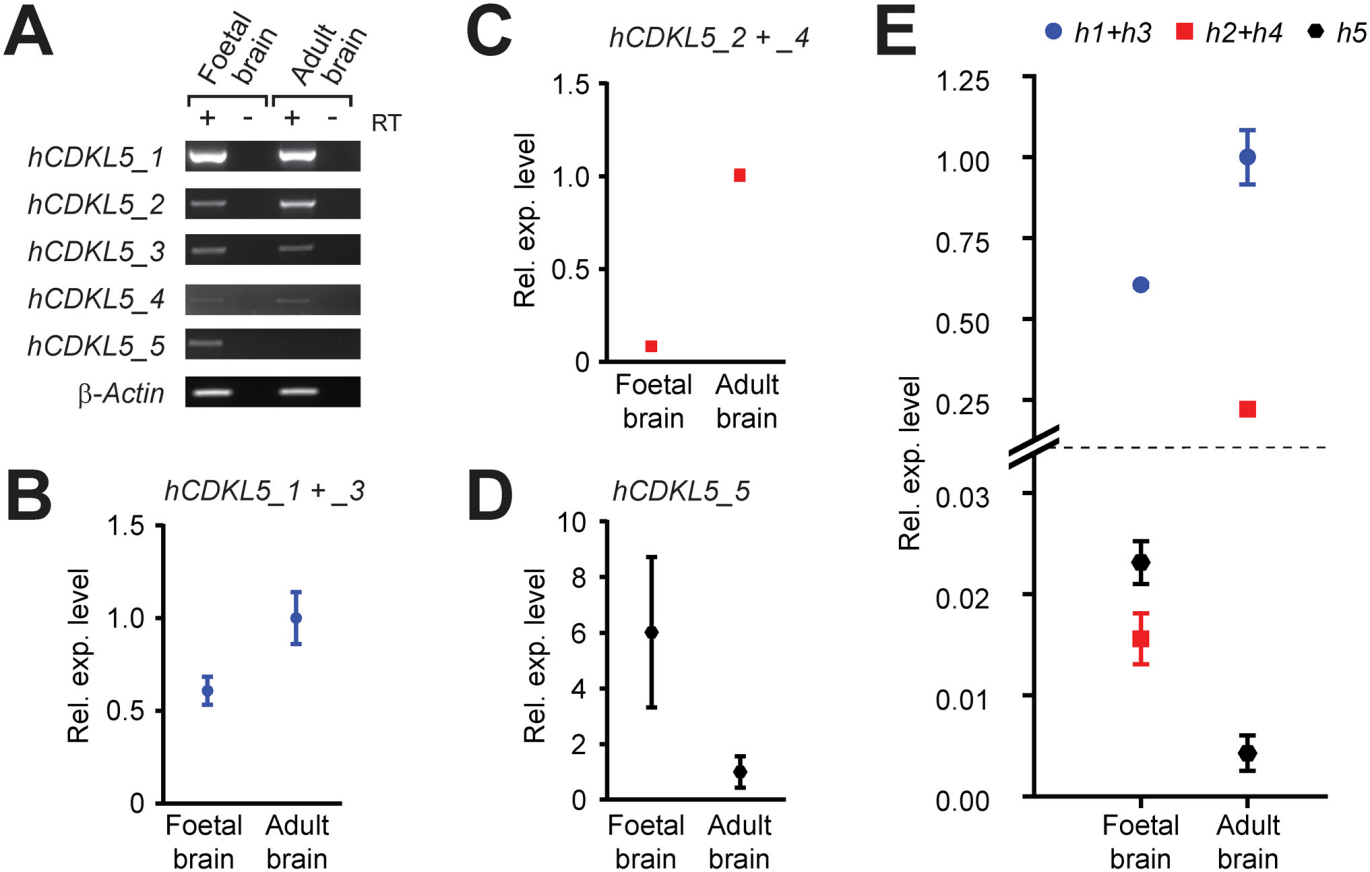
*CDKL5* expression levels during development. End-point (A) and quantitative RT-PCR analysis (B-D) of *CDKL5* transcript isoforms in total RNA isolated from human foetal and adult brain. Expression levels in each assay are shown relative the adult brain sample. (E) Comparative expression levels of human transcripts as revealed by 2^-ΔCt^ analysis are shown, where all values are relative to the *hCDKL5_1* + *_3* assay of the adult brain. All qRT-PCR assays were normalised to β-Actin endogenous controls and are shown as dot plots; bars indicate the standard error of the mean. It was not possible to discriminate between *hCDKL5_1* and *hCDKL5_3*, or *hCDKL5_2* and *hCDKL5_4* transcripts, due to qRT-PCR design constraints and therefore these results show the cumulative expression of both isoforms.

Mutations in *CDKL5* have neurodevelopmental consequences and therefore we wished to assess the expression of *CDKL5* transcripts during pre- and perinatal brain development. An analysis of *CDKL5* expression in the human brain during pre- and post-development was carried out using qRT-PCR and semi-quantitative end-point RT-PCR on a total RNA sample isolated from foetal brain and compared to the expression in adult whole brain. The CNS isoforms, *hCDKL5_1*, *_2*, *_3* and *_4*, are all expressed at higher levels in the adult brain than in the foetal brain, especially *hCDKL5_2* and *4* (Fig 7). Isoform *hCDKL5_5*, which is expressed only in the testis in the adult (Fig 2) is, however, expressed in the foetal brain (Fig 7D and 7E).

**Fig 8:**
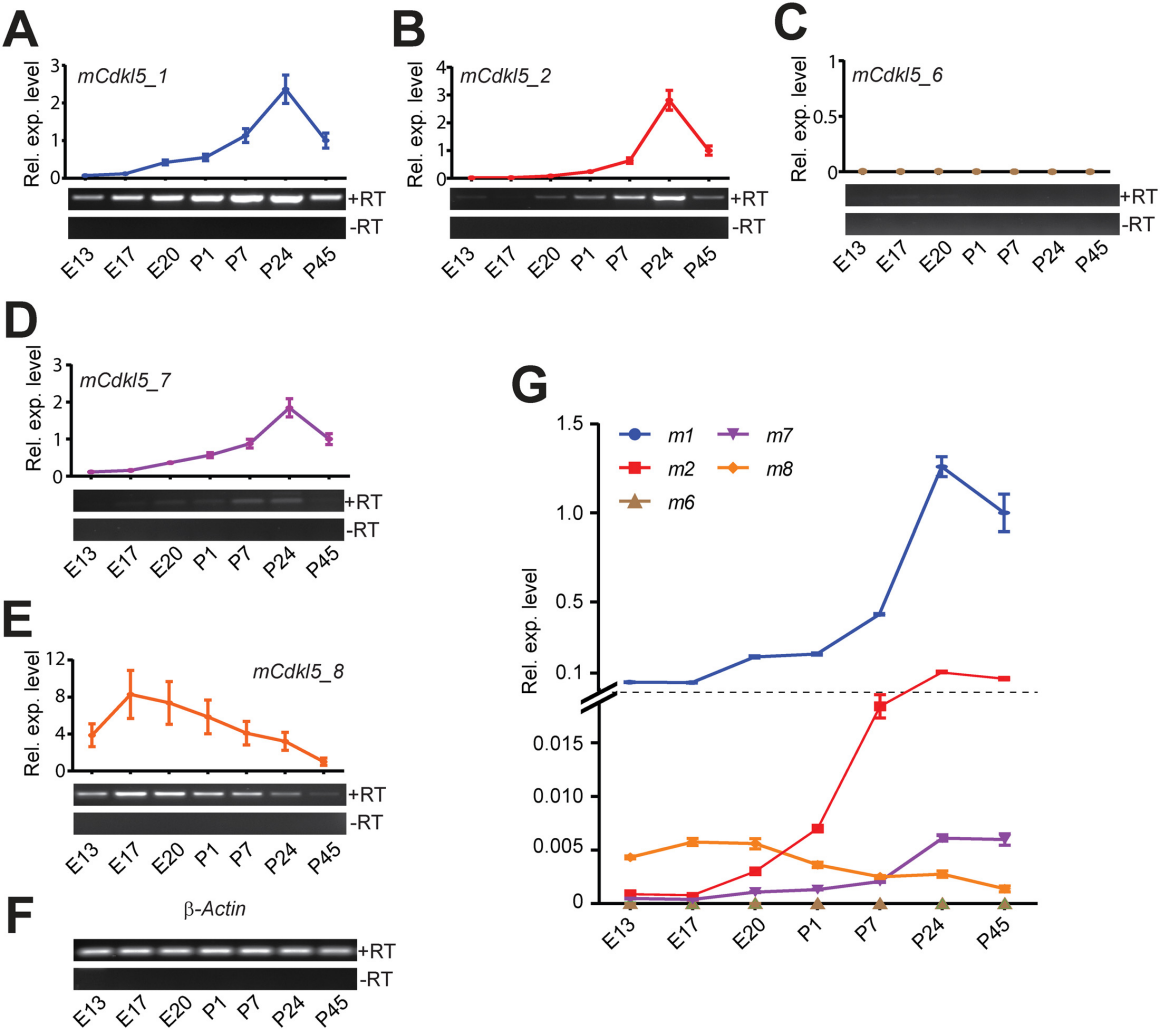
*Cdkl5* expression levels during development. (A-E) End-point and quantitative RT-PCR analysis of *Cdkl5* transcript isoforms in total RNA isolated from mouse whole brains at different times of embryonic (E13-E20) and postnatal (P1–P45) development. Three animals were analysed at each time-point; one representative sample from each time-point is shown on the gel image. Expression levels in each assay are shown relative the P45 sample. (F) β-actin loading control. (G) Comparative expression levels of the mouse qRT-PCR assays using 2^-ΔCt^ analysis are shown, where all values are relative to the *mCdkl5_1* assay of the P45 adult brain. All qRT-PCR assays were normalised to β-Actin endogenous controls and are shown as dot plots; bars indicate the standard error of the mean.

The expression of *Cdkl5* across a developmental time series in mouse was also investigated. Total RNA was isolated from the brains of mouse embryos at embryonic day 13, 17 and 20, and postnatal days 1, 7, 24 and 45 (n = 3 at each time-point). RT-PCR and qRT-PCR analysis showed that *mCdkl5_1* and *mCdkl5_2* are expressed throughout embryonic and early postnatal development, increasing in levels and peaking within the first few weeks after birth, with *mCdkl5_2* being expressed at lower levels than *mCdkl5_1* (Fig 8). Of the two isoforms expressed predominantly in the adult mouse testis, *mCdkl5_6* was undetectable in the brain at any time-point and *mCdkl5_7* was detected only at very low levels in the P7, P24 and P45 brain (Fig 8C and 8D). *mCdkl5_8*, which is expressed at low levels in the adult mouse brain, is expressed throughout development, showing maximal expression at late embryonic stages E17 and E20 and decreasing thereafter to adulthood (Fig 7E). In terms of relative abundance, a comparison across transcripts revealed pronounced differences in expression profiles over time (Figs 7E & 8G, for human and mouse respectively).

## Discussion

CDKL5 disorder is a rare, debilitating form of early infantile epileptic encephalopathy and severe intellectual disability caused by a range of *de novo* mutations in the *CDKL5* gene. Understanding how these mutations affect CDKL5 function requires a detailed knowledge of the gene structure and isoform expression. Using a combination of bioinformatic analyses and molecular methods we extend previous descriptions of the gene structure of *CDKL5* [5, 24, 25, 32, 33] to provide a detailed characterisation of the human and mouse *CDKL5* transcript sets, permitting a detailed cross-species comparison. We have confirmed previous findings that *hCDKL5_1* (formerly *CDKL5*_107_) is the predominant brain isoform of *CDKL5*. *hCDKL5_1* is a 9.7 kb transcript comprised of 18 exons and a large 6.6 kb 3’-UTR. However, we now demonstrate considerably greater diversity in human and mouse *CDLK5* transcripts than previously realised. This diversity arises from alternative promoter and first exon usage and from alternative splicing, and these patterns are in many cases species-specific.

### Cross-Species Comparison in *CDKL5* Isoform Expression

**Fig 9:**
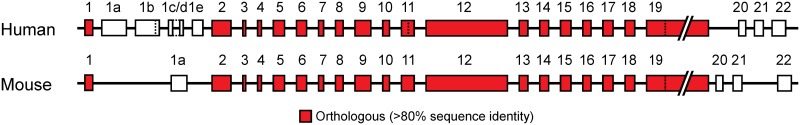
Comparison of human *CDKL5* and mouse *Cdkl5* gene structures.

We identified all splicing events that occur across a diverse panel of tissues and validated these by Sanger sequencing of RT-PCR products spanning multiple exons. This thereby elucidated even rare splice variants in both mouse and human brain tissue. The majority of the *CDKL5* coding region is orthologous and well-conserved between human and mouse (Fig 9). There are, however, exonic regions towards the 5’ end and 3’ end of each gene that show less conservation (29–42% sequence identity across the equivalent genomic DNA sequence) and are involved in the generation of alternative transcripts and several different CDKL5/Cdkl5 C-terminal regions. In total, five different coding transcript isoforms in human *CDKL5* and five in mouse *Cdkl5* were characterised. However, only the two isoforms expressed most abundantly in the CNS, *hCDKL5_1*/*mCdkl5_1* and *hCDKL5_2*/*mCdkl5_2*, were found to be completely orthologous (Table 3).

Our results confirm that *hCDKL5_1* (formerly *CDKL5*_107_) is the most abundant transcript isoform in the CNS for both mouse and human [25]. This isoform was detected in all other tissues tested, but at lower levels. Previous northern blot data had suggested that very large *CDKL5*/*Cdkl5* transcripts were expressed in human and mouse brains, and that a different, smaller transcript is expressed in human and mouse testes [24]. However, this preliminary observation had not been followed up in subsequent studies. Our characterisation of *CDKL5* UTRs confirms that, in adults, large transcripts of ~9.7 and ~12.8 kb in human, and ~9.7 kb in mouse, are predominantly expressed in the CNS; and that transcripts of ~3.4 kb in human and ~3.2 kb in mouse are almost exclusively expressed in the testis.

In addition to identifying this variation, we looked for evidence confirming the existence of theoretical transcripts *hCDKL5*_*115+ex*.*16b*_ and *hCDKL5*_*-ex*.*19m*_ predicted by previous studies [17], but found none. Although the full significance of this transcript diversity is unclear, it is apparent that the putative catalytic domain of CDKL5 is preserved across all isoforms and this corresponds to a region within which most pathogenic missense mutations occur [17, 29]. The species-specific diversity identified occurs within regulatory regions (5’ and 3’) and at the very end of the C-terminal domain of CDKL5. This correlates with regions of the gene that show the lowest degree of homology, suggesting that the observed alternative splicing may be important in regulating expression and function. Variation in regions outside the catalytic domain (exons 17 and part of exon 11) is also seen (Fig 1, S3 Fig). Exon 17 is present in transcripts *hCDKL5_2* and *hCDKL5_4* and its inclusion adds 41 amino acids to the protein. This exon is extremely well conserved between human and mouse, but we can find no evidence of it containing known functional elements. Use of the cryptic splice site in exon 11 (*hCDKL5_3* and *hCDKL5_4*) results in the loss of 17 amino acids, a region that contains a putative nuclear localisation signal [43], suggesting that human isoforms utilising this splice site may lead to altered ratios of CDKL5 protein in different cellular compartments. The functional relevance of these isoforms is at present unknown, but, in terms of expression levels, they account for less than 10% of *CDKL5* transcripts expressed in whole brain (Fig 7, S3 Fig).

It will be important to examine *CDKL5*/CDKL5 expression in human tissue to determine whether human-specific isoforms show restricted spatial and temporal expression profiles, as this may yield insights into the targeting of mRNA/protein to discrete cellular compartments. However, currently, the lack of effective antibodies against CDKL5 has hampered efforts to fully characterise the various protein isoforms.

### *CDKL5* Complexity: Promoters, TSSs and UTRs

As discussed above, diversity between species was particularly prominent in alternative promoter and first exon usage. The 5’-UTR shows far greater complexity in humans than in mouse. Little is known about transcriptional regulation of *CDKL5* and its various putative promoter regions remain poorly defined. In a recent study by the FANTOM5 project, *CDKL5* was predicted to contain a TATA-less promoter lying within a CpG island, in a region immediately upstream of exon 1 [44]. The major TSS in both human and mouse mapped to the same nucleotide in our 5’-RACE analysis, in agreement with the DataBase of Transcriptional Start Sites [45]. A number of other TSSs were found to cluster close by, but not in a ‘broad’ distribution as expected for genes with a CpG islands (Fig 3A) [46]. This ‘intermediate’-type promoter may have consequences for the regulation of *CDKL5* expression, potentially via downstream transcription-associated chromatin organisation [47]. We have identified multiple first exons and we therefore predict that four further promoters lying between exons 1 and 2 should exist. Further studies are required to characterise these additional promoters.

The identification of large 3’-UTRs (> 6.6 kb) in the major brain isoforms may suggest additional, previously unrecognised, modes of regulation of *CDKL5*. *mCdkl5* mRNA has been reported to be present in dendrites in the adult brain where it may have a role in local Cdkl5 synthesis [18]. It is possible that these large 3’-UTRs may have a role in trafficking *Cdkl5* to the dendrites, and it is noticeable that there is a high degree of sequence conservation within this region (Fig 9). However, an analysis of the potential role of large conserved UTRs in mRNA trafficking within neurons requires further investigation. A number of putative miRNA binding sites in the 3’-UTR have already been recognised [48, 49], but the importance of this mode of regulation is not clearly established for CDKL5, as a review of recent studies suggested that CDKL5 protein levels tend to correlate with mRNA expression levels in the adult brain [17].

### Temporal and Spatial Diversity of *CDKL5*

Our analysis of *CDKL5*/*Cdkl5* expression reveals a dynamic regulation of individual transcript levels during neurodevelopment. Indeed we find that transcripts *mCdkl5_8* and *hCDKL5_5*, previously believed to be testis-specific, are expressed in the brain during development. This finding is of particular significance since mutations in exons 20–22 have hitherto been classified as being non-pathogenic [50]. More generally, in the mouse brain, Cdkl5 protein expression has been reported to be at low levels during early developmental stages (E16.5), and then strongly induced during early postnatal stages [12]. Our results show examples of transcripts whose expression levels change in a more graded fashion over extended periods of development; for example, *mCdkl5_1* increases from E17-P24, whereas *mCdkl5_8* shows a gradual decrease over the same period. The detection of different transcript isoforms is suggestive of complex developmental regulation of *CDKL5* expression patterns and levels during pre- and postnatal development. However, the functional significance of different *CDKL5* transcript isoforms and whether there are levels of redundancy across *CDKL5* transcripts remains to be assessed.

### Nomenclature

To accompany the analysis of human *CDKL5* and mouse *Cdkl5* transcript isoforms a new standardised nomenclature system is proposed. In addition to the former names described in Table 3, designations such as ‘isoform I’ and ‘isoform II’ have also been used, and the number of alternative designations for the same entity has been an obstacle to consistent *CDKL5* terminology. The new proposed nomenclature system retains flexibility and takes into account the complexity of transcript isoforms characterised in this study, providing a reference for future work on this gene.

Overall, the complexity of *CDKL5* transcripts highlighted in this study needs to be taken into account when developing gene-based, protein-based or pharmacotherapies for CDKL5 disorders. The data may also aid the development of isoform-specific antibodies. The complexity and dynamic regulation of CDKL5 may also be important when considering the time-course of CDKL5 disorder pathophysiology and potential time-points for therapeutic intervention. The detailed characterisation of *CDKL5/Cdkl5* should not only inform the design of *CDKL5* mutation screening assays but, importantly, also provide a valuable platform for fundamental research into the biology of *CDKL5*.

## Supporting Information

S1 Fig Primer locations and sequences. PCR Primer pairs specific for each isoform in (A) human and (B) mouse are listed, and the diagrams indicate the coverage of the resultant amplicons. (C) The primer pair designed to amplify *hCDKL5_1* will also amplify *hCDKL5_5*, if *hCDKL5_5* is expressed in that tissue. As *hCDKL5_5* is expressed primarily in the adult testis, the assay is therefore quite specific for *hCDKL5_1*. An alternative primer pair that amplifies *hCDKL5_1* + *_3* simultaneously was tested in the panel of adult tissues and a similar pattern of expression observed.
(TIF)

S2 Fig *CDKL5* isoform expression in human tissues. RT-PCR analysis of *CDKL5* transcript isoforms in a panel of adult tissues. *GAPDH* was used as a loading control.
(TIF)

S3 Fig Alternative splicing affecting human *CDKL5* and mouse *Cdkl5* coding regions. RNA-seq data from brain (red) and testis (blue) datasets show reads mapping to (A) exons 16, 17 and 18 in human and mouse and (B) exons 10, 11 and 12 in human. In both diagrams the number on the y-axis indicates maximum read count and the dotted lines between splice donor and acceptor sites indicate the number of reads that map to that exon-exon junction. All reads contributing to these data span a maximum of two exons. (C) RT-PCR of total RNA isolated from brain tissue in human and mouse. Products confirming the presence of exon 17 in *CDKL5* and *Cdkl5* transcripts were gel-purified and sequenced (the 285 bp band in the left gel image). A low abundance product confirming the use of an alternative splice site in exon 11 in *CDKL5* transcripts was gel-purified and sequenced (the 146 bp band in the right gel image). (D,E) Sequence of PCR amplicons in (C). Primers are indicated by red arrows. The 3’ end of exon 11 in human and mouse, showing the presence of a cryptic splice site in human and its absence in mouse due to a single nucleotide difference (highlighted in red).
(TIF)
