# Supplementary figures and images for: Characterisation of CDKL5 Transcript Isoforms in Human and Mouse
Source: PLoS One. 2016 Jun 17;11(6):e0157758. doi: 10.1371/journal.pone.0157758 (PMC4912119; doi:10.1371/journal.pone.0157758)

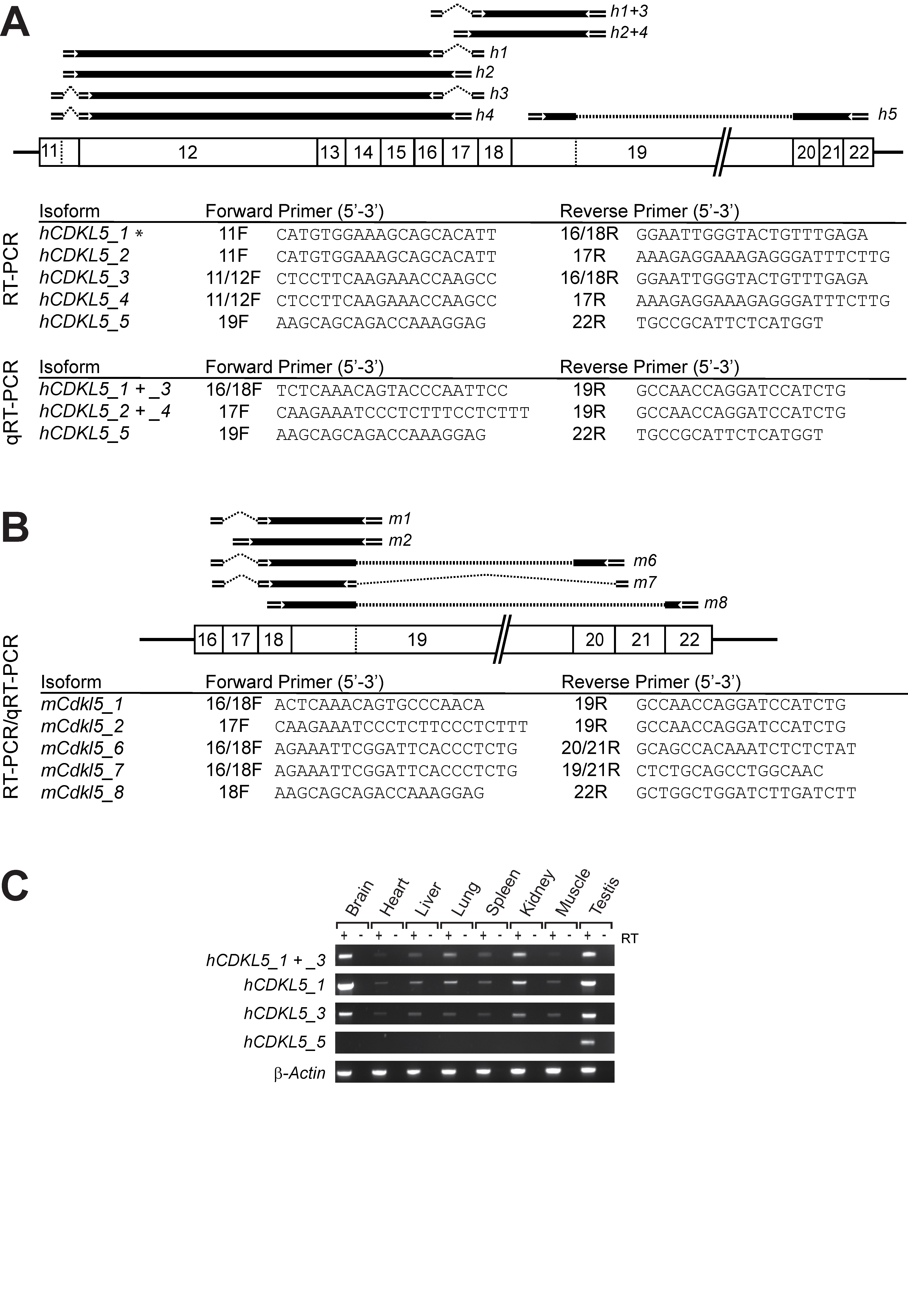

Supplement: S1 Fig — PCR Primer pairs specific for each isoform in (A) human and (B) mouse are listed, and the diagrams indicate the coverage of the resultant amplicons. (C) The primer pair designed to amplify hCDKL5_1 will also amplify hCDKL5_5, if hCDKL5_5 is expressed in that tissue. As hCDKL5_5 is expressed primarily in the adult testis, the assay is therefore quite specific for hCDKL5_1. An alternative primer pair that amplifies hCDKL5_1 + _3 simultaneously was tested in the panel of adult tissues and a similar pattern of expression observed. (TIF) [file pone.0157758.s001.tif]

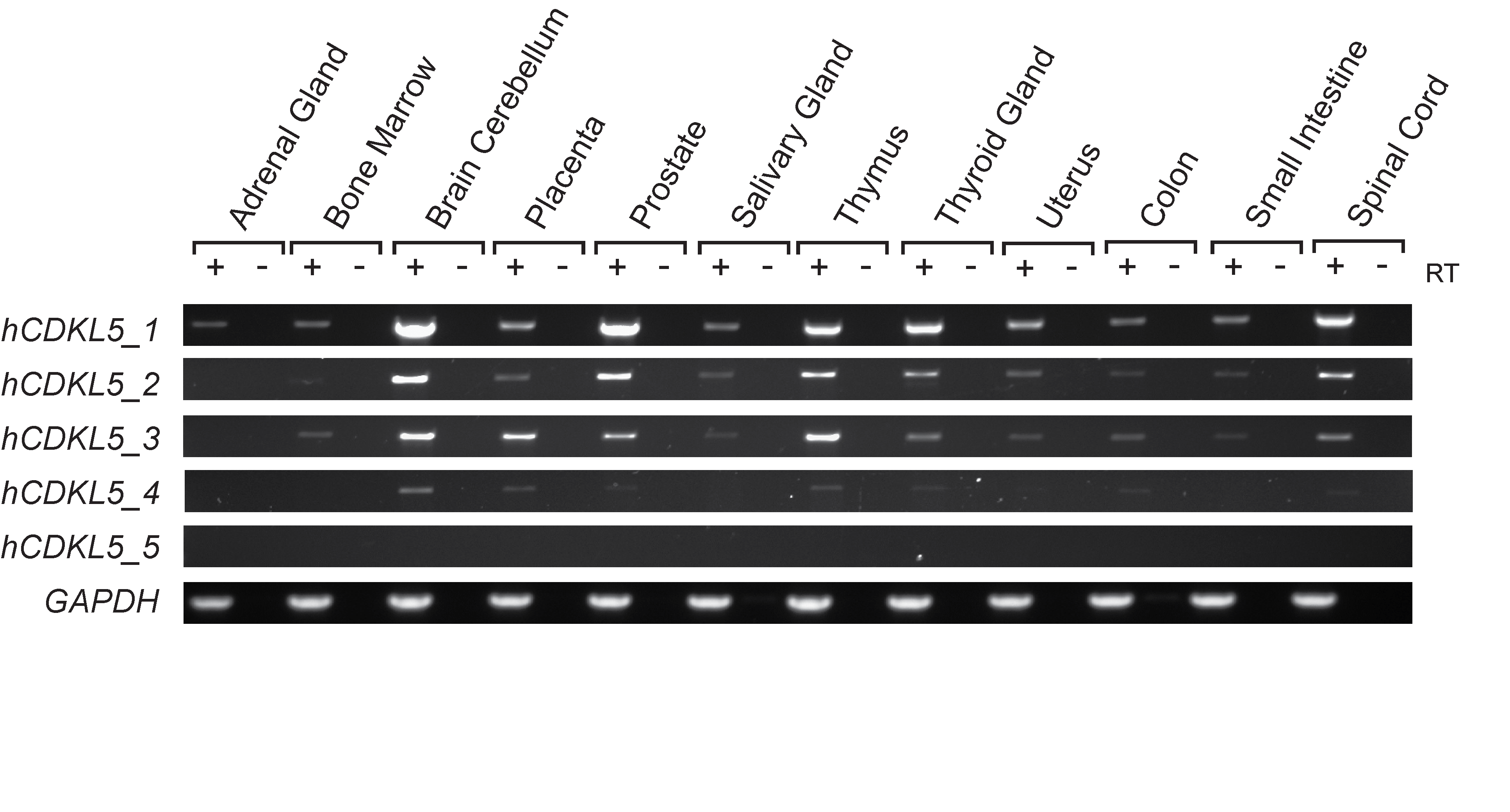

Supplement: S2 Fig — RT-PCR analysis of CDKL5 transcript isoforms in a panel of adult tissues. GAPDH was used as a loading control. (TIF) [file pone.0157758.s002.tif]

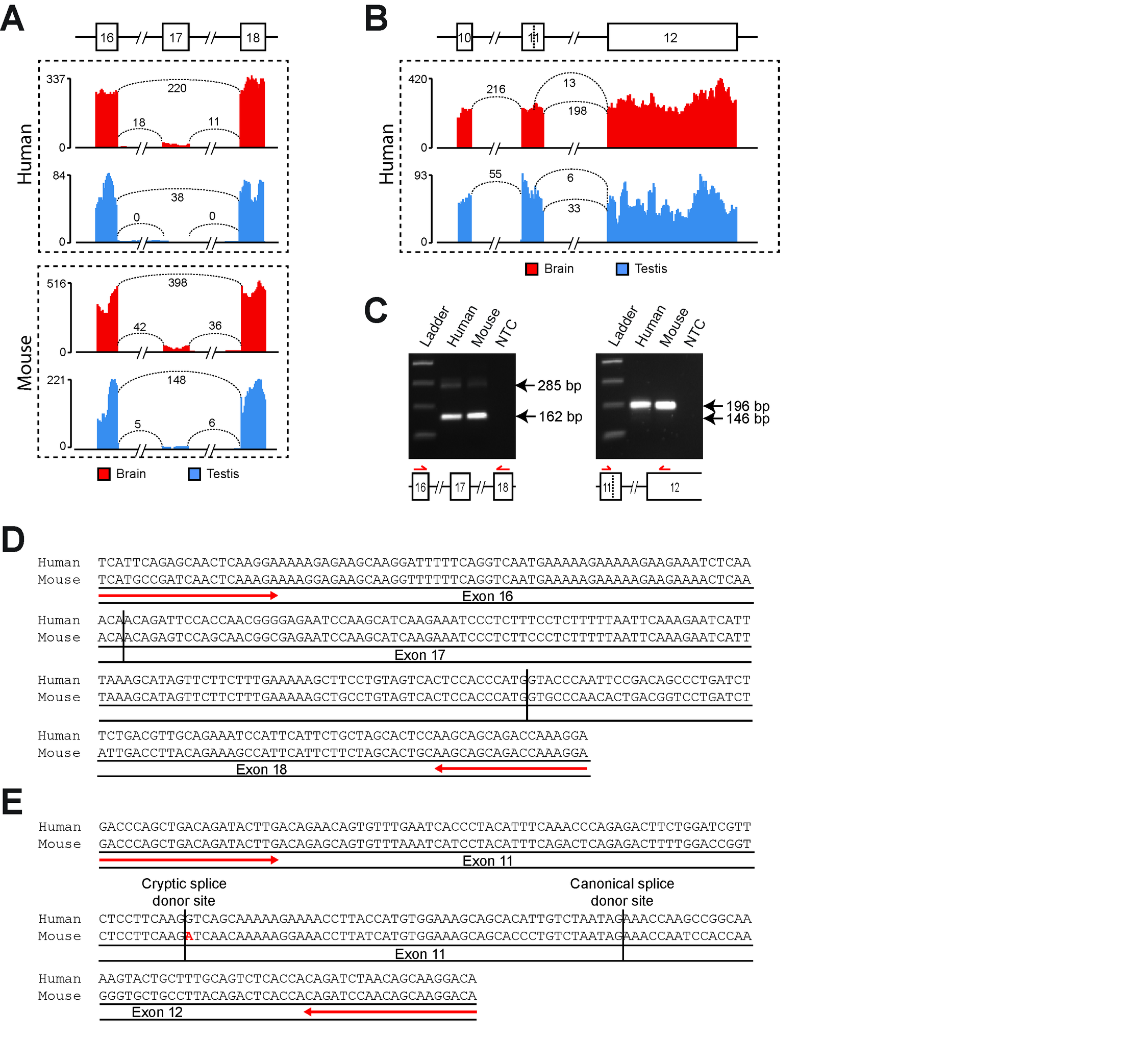

Supplement: S3 Fig — RNA-seq data from brain (red) and testis (blue) datasets show reads mapping to (A) exons 16, 17 and 18 in human and mouse and (B) exons 10, 11 and 12 in human. In both diagrams the number on the y-axis indicates maximum read count and the dotted lines between splice donor and acceptor sites indicate the number of reads that map to that exon-exon junction. All reads contributing to these data span a maximum of two exons. (C) RT-PCR of total RNA isolated from brain tissue in human and mouse. Products confirming the presence of exon 17 in CDKL5 and Cdkl5 transcripts were gel-purified and sequenced (the 285 bp band in the left gel image). A low abundance product confirming the use of an alternative splice site in exon 11 in CDKL5 transcripts was gel-purified and sequenced (the 146 bp band in the right gel image). (D,E) Sequence of PCR amplicons in (C). Primers are indicated by red arrows. The 3’ end of exon 11 in human and mouse, showing the presence of a cryptic splice site in human and its absence in mouse due to a single nucleotide difference (highlighted in red). (TIF) [file pone.0157758.s003.tif]
